# Supplementary material for: Online Bayesian Phylodynamic Inference in BEAST with Application to Epidemic Reconstruction
Source: Mol Biol Evol. 2020 Feb 26;37(6):1832–42. doi: 10.1093/molbev/msaa047 (PMC7253210; doi:10.1093/molbev/msaa047)
Supplement: msaa047_Supplementary_Data [file msaa047_supplementary_data.zip › msaa047-suppl_data/suppmat.pdf]

Supplementary material for "Online Bayesian phylodynamic inference in BEAST with application to epidemic reconstruction"

Mandev S. Gill, Philippe Lemey, Marc A. Suchard, Andrew Rambaut, and Guy Baele

1 Online Inference Methodology

1.1 Sequence insertion

Algorithm S1: Sequence insertion approach

```
newSeq ← newly available sequences;
tree ← tree with previously available sequences;
ϵ ← number in (0, 1);
for each sequence ∈ newSeq do
    closest ← getClosestSequence(sequence, tree);
    timeForDistance ← getDistance(sequence, closest)/getEvolutionaryRate(closest);
    timeClosest ← getDate(closest);
    timeNew ← getDate(sequence);
    timeGreater ← max(timeClosest, timeNew)
    timeInsert ← timeGreater + (timeForDistance − |timeClosest − timeNew|)/2;
    if timeInsert ≤ timeGreater then
        timeInsert ← timeGreater + ϵ(getDate(getParent(closest)) − timeGreater);
    end if
    splitChild ← closest;
    if timeInsert ≥ getDate(getParent(splitChild)) then
        parent ← getParent(splitChild);
        while timeInsert ≥ getDate(parent) do
            if isRoot(parent) or timeInsert = getDate(parent) then
                timeInsert ← getDate(splitChild) + ϵ ∗ (getDate(parent) − getDate(splitChild));
                break;
            else
                splitChild ← parent;
                parent ← getParent(splitChild);
            end if
        end while
    end if
    tree ← insertSequenceInTree(sequence, timeInsert, splitChild, tree);
    updateRateCategories(tree, sequence);
end for
return tree;
```

1.2 Imputing branch rates under an uncorrelated relaxed molecular clock model

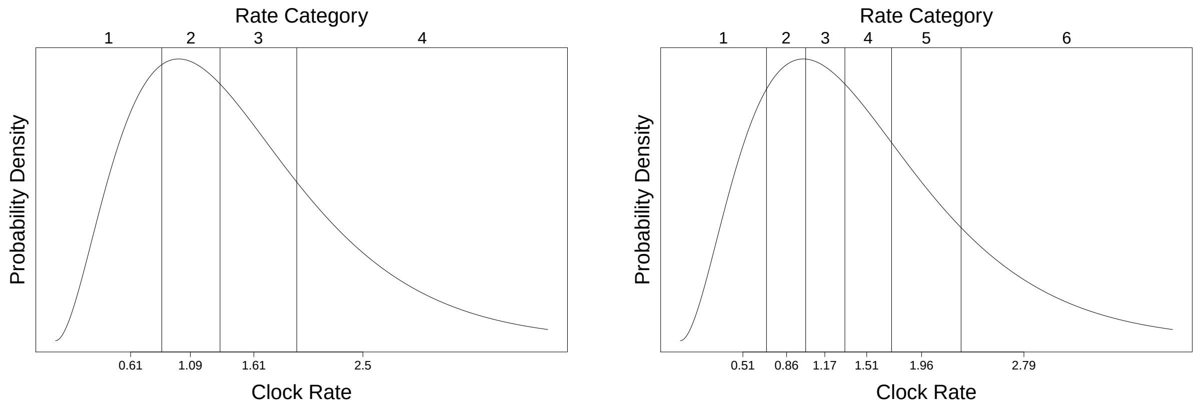

Figure S1: Discretization of gamma distributions into rate categories for uncorrelated relaxed molecular clock rates. The left figure shows a discretization into $n = 4$ categories, while the right figure shows a discretization of the same distribution into $n = 6$ categories, after a new sequence has been inserted into the tree, adding two new branches and two new rate categories to the discretized distribution.

The uncorrelated relaxed molecular clock model (Drummond et al., 2006) posits that branch-specific clock rates are drawn independently from an underlying rate distribution (e.g., a gamma or log-normal distribution) that is discretized into a number of categories equal to the number of tree branches. Let cat(n,i) denote the kth rate category under an uncorrelated relaxed clock with n categories. Then under the discretization, each of the n categories has equal probability $1/n$, and cat(n,k) corresponds to the $(k - 0.5)/n$ quantile of the rate distribution, as shown in Figure S1. To integrate branch rates out, Drummond et al. (2006) associate each of the n branches of the tree with one of the n rate categories (multiple branches can correspond to the same rate category) and use MCMC to sample the assignment of rate categories to branches.

Note that the growth of a tree after insertion of a new sequence means that each of its branches will assume a new clock rate, even if it retains the same category. However, the aim of our online inference procedure is to assign branches in the enlarged tree rate categories equal to or close to the categories they assumed before the tree was enlarged, ensuring that the clock rates assumed by branches are relatively close to the clock rates they assumed before the analysis was interrupted.

We describe the imputation procedure here, and it is also illustrated in Figure S2 and Algorithm S2. The procedure begins by assigning branches in the enlarged tree that lead to nodes that existed in the tree before it was enlarged the same rate categories they previously assumed. After insertion of a sequence, the enlarged tree features two new branches: the “first” new branch, which leads to the new sequence, and the “second” new branch, which leads to the new ancestral node. The first new branch is assigned the same rate category as its sibling branch, incremented by one. Then, all branches in the tree that assume rate categories greater than or equal to the rate category of the first new branch have their rate categories incremented by one. This is to make use of the additional rate categories that are available after the tree has been enlarged, while still allowing the branches that already existed in the tree to retain rates close to their previous values. Next, the second new branch is assigned the same rate category as its sibling branch. Then, all branches in the tree that assume rate categories greater than or equal to the rate category of the second new branch have their rate categories incremented by one.

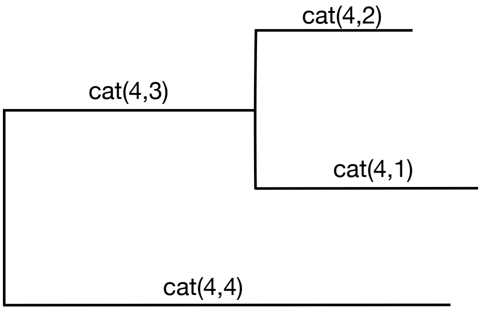

(a) Initial tree annotated with rate categories.

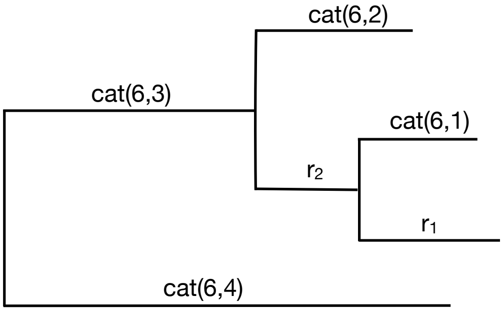

(b) Expanded tree after insertion of new sequence. Branches leading to nodes that were in initial tree inherit rate categories from corresponding branches in initial tree.

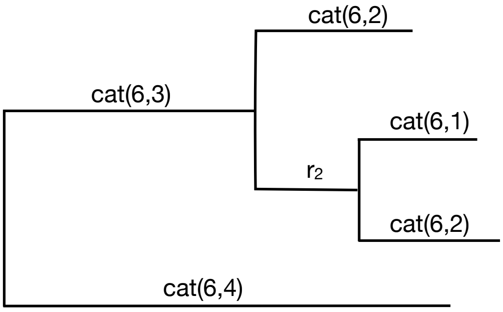

(c) Missing rate category r_1 is imputed by taking rate category for sibling branch and incrementing by one.

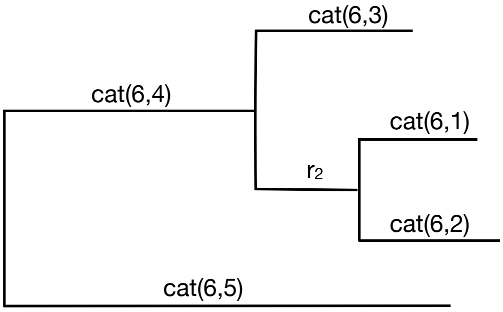

(d) Rate categories for other branches are incremented by one if they are greater than or equal to $\mathrm{r}_1 = 2$.

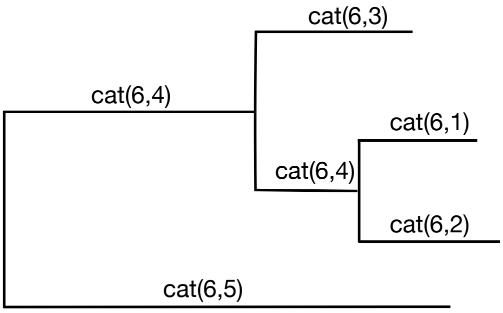

(e) Missing rate category for r_2 is imputed by taking rate category for sibling branch and incrementing by one.

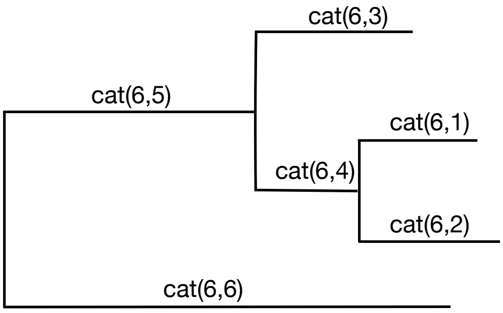

(f) Rate categories for other branches are incremented by one if they are greater than or equal to $\mathrm{r}_2 = 4$.

Figure S2: Algorithm for imputing uncorrelated relaxed molecular clock rate categories after the phylogenetic tree has been enlarged by insertion of new sequence. Rate category k for a discretized distribution with n total categories is denoted $\mathrm{cat}(n,k)$. In this example, the initial tree (a) has a unique rate category associated with each branch. However, branches need not assume unique rate categories. Branch rate category assignments in the initial tree are from the random draw $\boldsymbol{\theta}_i$ taken from the posterior sample generated at step i, where $\boldsymbol{\theta}_i$ is modified by the online inference algorithm to generate a starting value $\boldsymbol{\theta}_{i+1}^{(0)}$ for the analysis of the expanded data set at step $i+1$ (see Materials and Methods).

Algorithm S2: Imputing branch rates under an uncorrelated relaxed molecular clock model

```
tree ← current augmented tree, featuring new branches after sequence insertion;
newTaxon ← taxon corresponding to newly inserted sequence;
firstNewBranch ← getBranchAssociatedWithTaxon(newTaxon);
secondNewBranch ← getParentBranch(firstNewBranch);
setBranchRateCategory(firstNewBranch, getBranchRateCategory(getSiblingBranch(firstNewBranch))+
1);
for each otherBranch ∈ tree do
    if otherBranch != firstNewBranch and otherBranch != secondNewBranch then
        if getBranchRateCategory(otherBranch) ≥ getBranchRateCategory(firstNewBranch)
        then
            setBranchRateCategory(otherBranch, getBranchRateCategory(otherBranch) + 1);
        end if
    end if
end for
setBranchRateCategory(secondNewBranch, getBranchRateCategory(getSiblingBranch(secondNewBranch))+
1);
for each otherBranch ∈ tree do
    if otherBranch != secondNewBranch then
        if getBranchRateCategory(otherBranch) ≥ getBranchRateCategory(secondNewBranch)
        then
            setBranchRateCategory(otherBranch, getBranchRateCategory(otherBranch) + 1);
        end if
    end if
end for
return tree;
```

2 Performance

2.1 Comparison of burn-in for analyses of Ebola virus data set

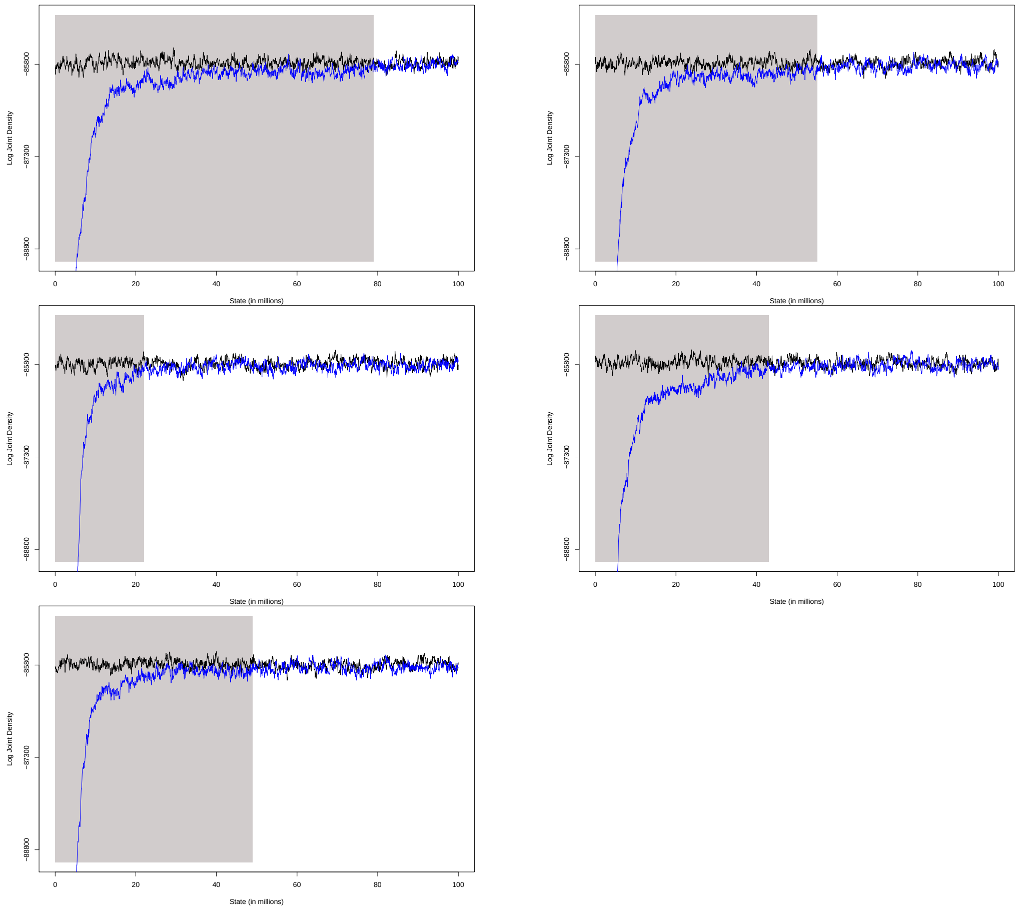

Figure S3: Comparison of burn-in resulting from standard Bayesian inference versus online Bayesian inference for a large-scale Ebola virus data set, focusing on epi week 42 of 2015. The data set comprises 1610 complete genome Ebola virus sequences. Within each plot, the trace of the log joint (likelihood $\times$ prior) density resulting from a standard analysis is blue and the corresponding trace resulting from an online analysis, that updates inferences from the previous epi week upon the arrival of new data, is black. The shaded light gray regions in the background depict the burn-in for the standard analyses (no visible burn-in for the online analyses).

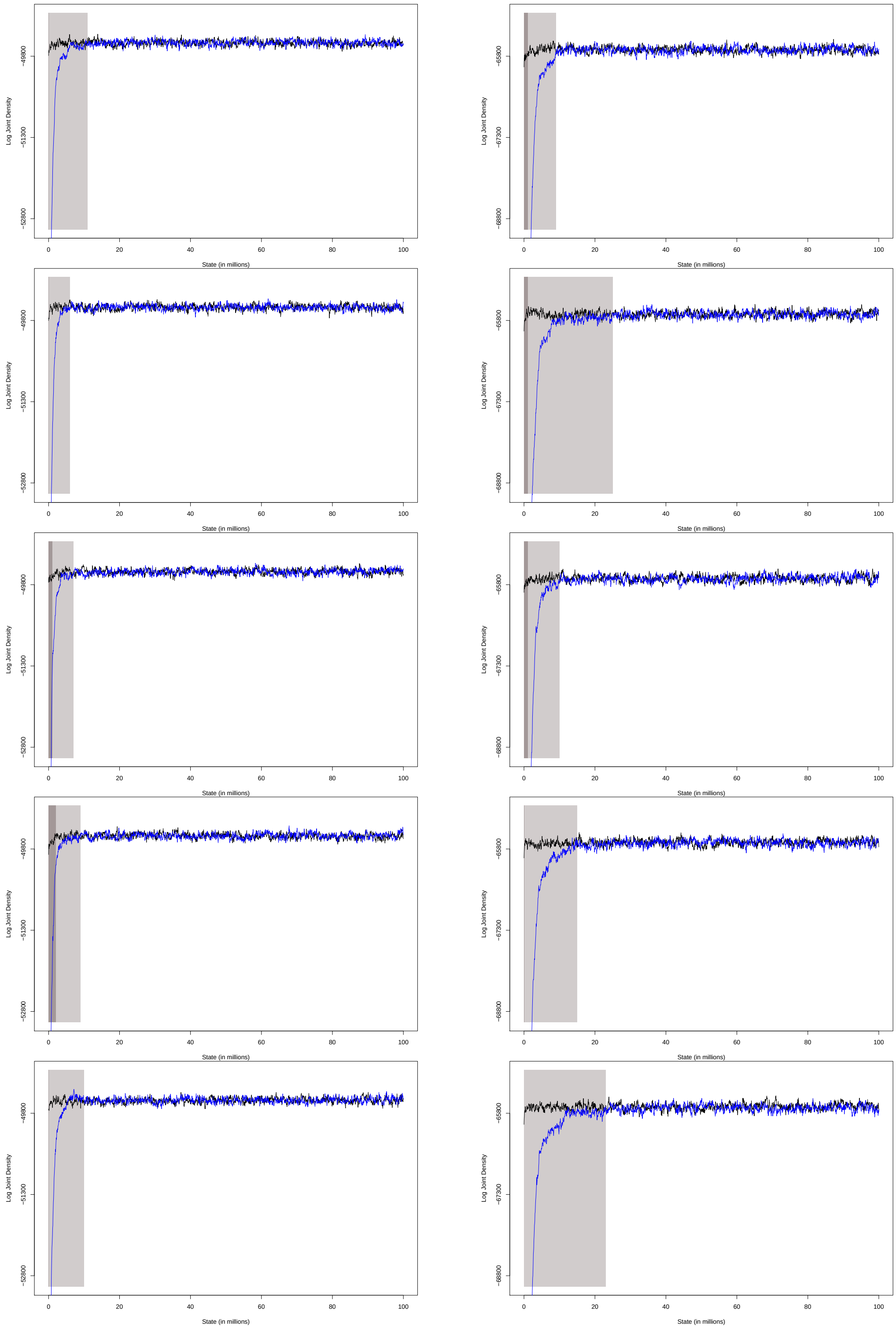

Figure S4: Comparison of burn-in resulting from standard Bayesian inference versus online Bayesian inference for a large-scale Ebola virus data set, focusing on epi week 42 of 2014 (column 1) and epi week 2 of 2015 (column 2). The data sets comprise 706 and 1072 complete genome Ebola virus sequences, respectively. Within each plot, the trace of the log joint density resulting from a standard analysis is blue and the corresponding trace resulting from an online analysis, that updates inferences from the previous epi week upon the arrival of new data, is black. The shaded light and dark gray regions in the background depict the burn-in (if any) for the standard and online analyses, respectively.

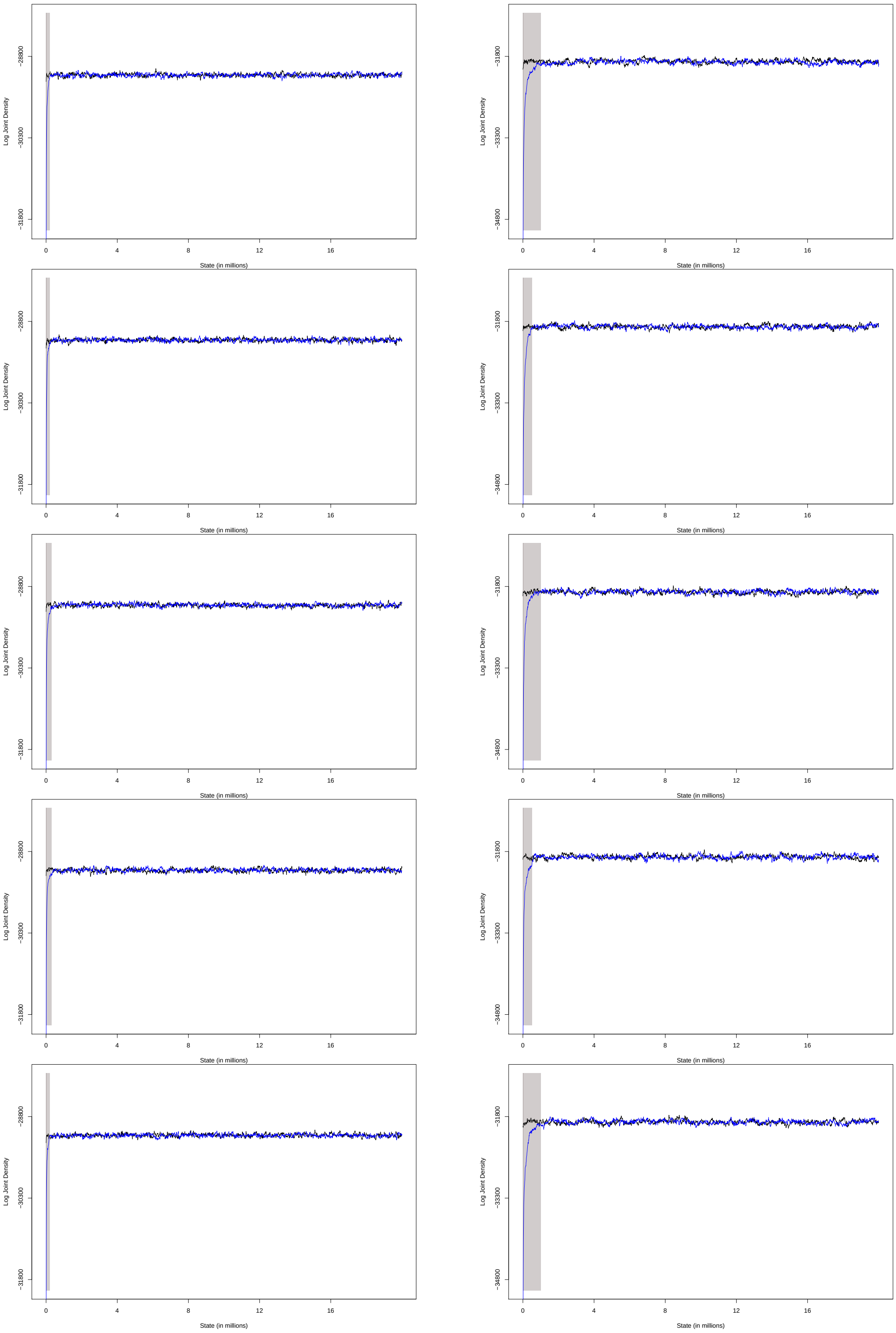

Figure S5: Comparison of burn-in resulting from standard Bayesian inference versus online Bayesian inference for a large-scale Ebola virus data set, focusing on epi weeks 26 (column 1) and 31 (column 2) of 2014. The data sets comprise 158 and 240 complete genome Ebola virus sequences, respectively. Within each plot, the trace of the log joint density resulting from a standard analysis is blue and the corresponding trace resulting from an online analysis, that updates inferences from the previous epi week upon the arrival of new data, is black. The shaded light gray regions in the background depict the burn-in for the standard analyses.

2.2 Comparison of tree samples for the Ebola virus analyses

To examine whether the independent replicates for a given time point in the Ebola virus epidemic converge to the same stationary distribution, we compare the frequencies of splits (or clades) across multiple independent Markov chains. In particular, we compare chains generated by the same method (standard inference or online inference) and by different methods by considering all possible pairwise comparisons for chains corresponding to the same data set. For each chain, we first remove the burn-in samples, and we then extract a subsample of 200 trees. The trees correspond to every 50,000th iteration for epi weeks 26 and 31 of 2014, and to every 250,000th iteration for the other epi weeks. Using the RWTY (R We There Yet) software package (Warren et al., 2017), we create plots of split frequencies for each pair of chains and also compute their correlation and the average standard deviation of split frequences (ASDSF) (Lakner et al., 2008). Results are shown in Figures S6-S10. As the different chains converge to the same stationary distribution, the ASDSF should approach 0. We adopt the guidelines that an ASDSF less than 0.05 (ideally, less than 0.01) supports topological convergence (Ronquist et al., 2011).

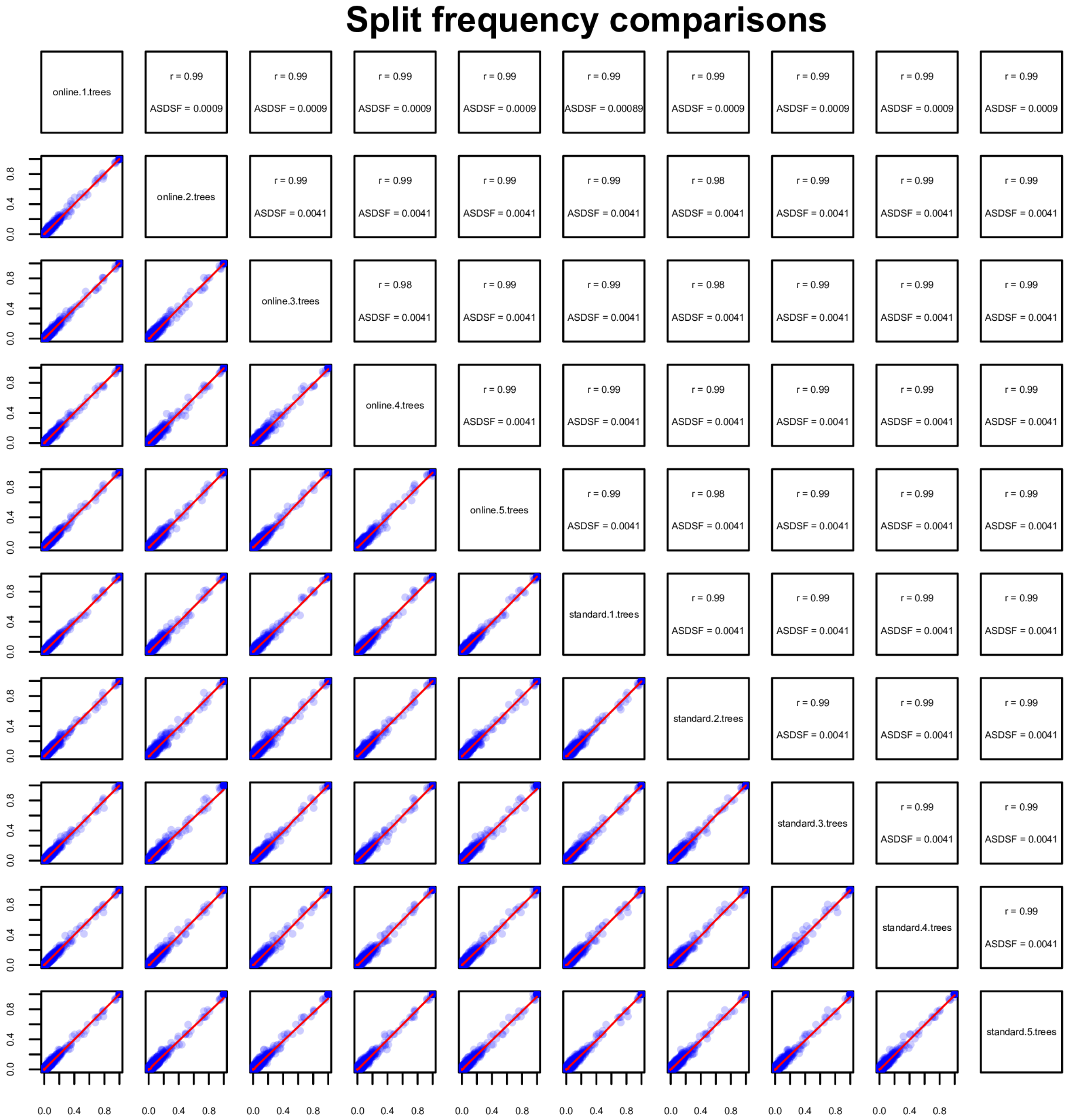

Figure S6: Comparison of split (clade) frequencies in tree samples from five standard and five online analyses of data from epi week 26 of 2014 of West African Ebola virus epidemic. For each pair of chains, the frequencies of clades in the different chains are plotted against each other in the plots below the diagonal of the figure, and the correlation and the average standard deviation of split frequencies (ASDSF) of the pair are shown above the diagonal. The diagonal entries correspond to the ten independent replicates and indicate the specific pairwise comparisons that are made below and above the diagonal.

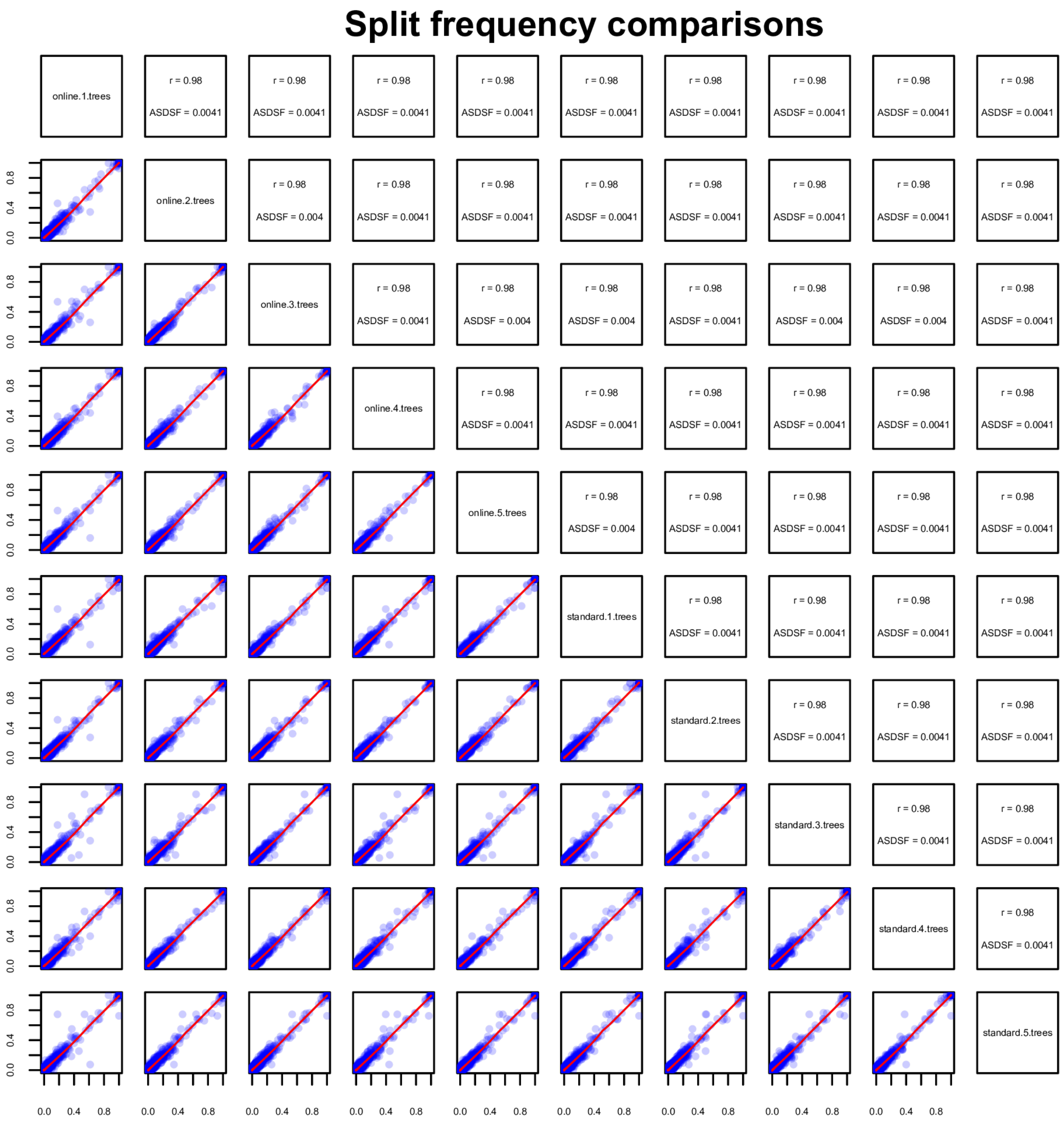

Figure S7: Comparison of split (clade) frequencies in tree samples from five standard and five online analyses of data from epi week 31 of 2014 of West African Ebola virus epidemic. For each pair of chains, the frequencies of clades in the different chains are plotted against each other in the plots below the diagonal of the figure, and the correlation and the average standard deviation of split frequencies (ASDSF) of the pair are shown above the diagonal. The diagonal entries correspond to the ten independent replicates and indicate the specific pairwise comparisons that are made below and above the diagonal.

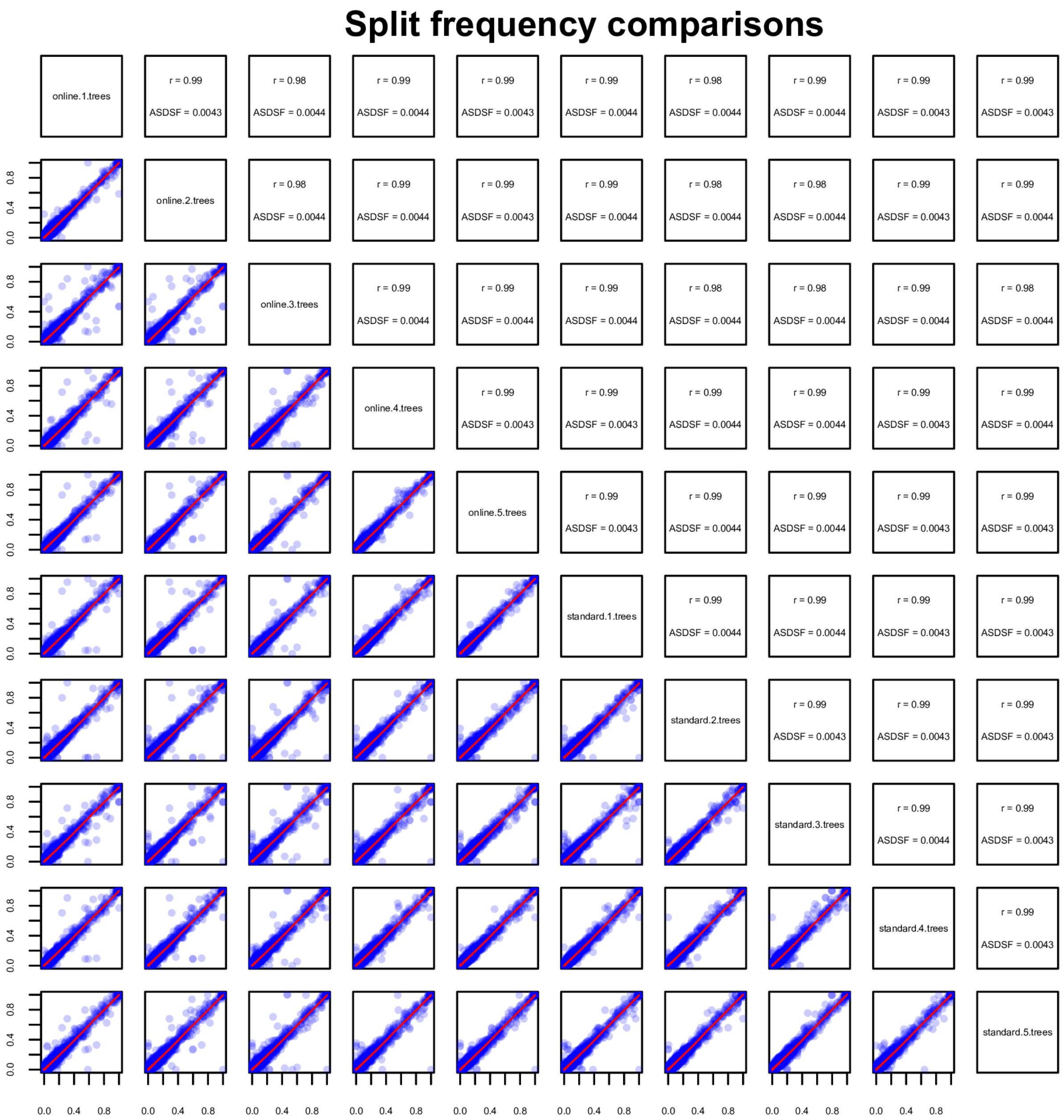

Figure S8: Comparison of split (clade) frequencies in tree samples from five standard and five online analyses of data from epi week 42 of 2014 of West African Ebola virus epidemic. For each pair of chains, the frequencies of clades in the different chains are plotted against each other in the plots below the diagonal of the figure, and the correlation and the average standard deviation of split frequencies (ASDSF) of the pair are shown above the diagonal. The diagonal entries correspond to the ten independent replicates and indicate the specific pairwise comparisons that are made below and above the diagonal.

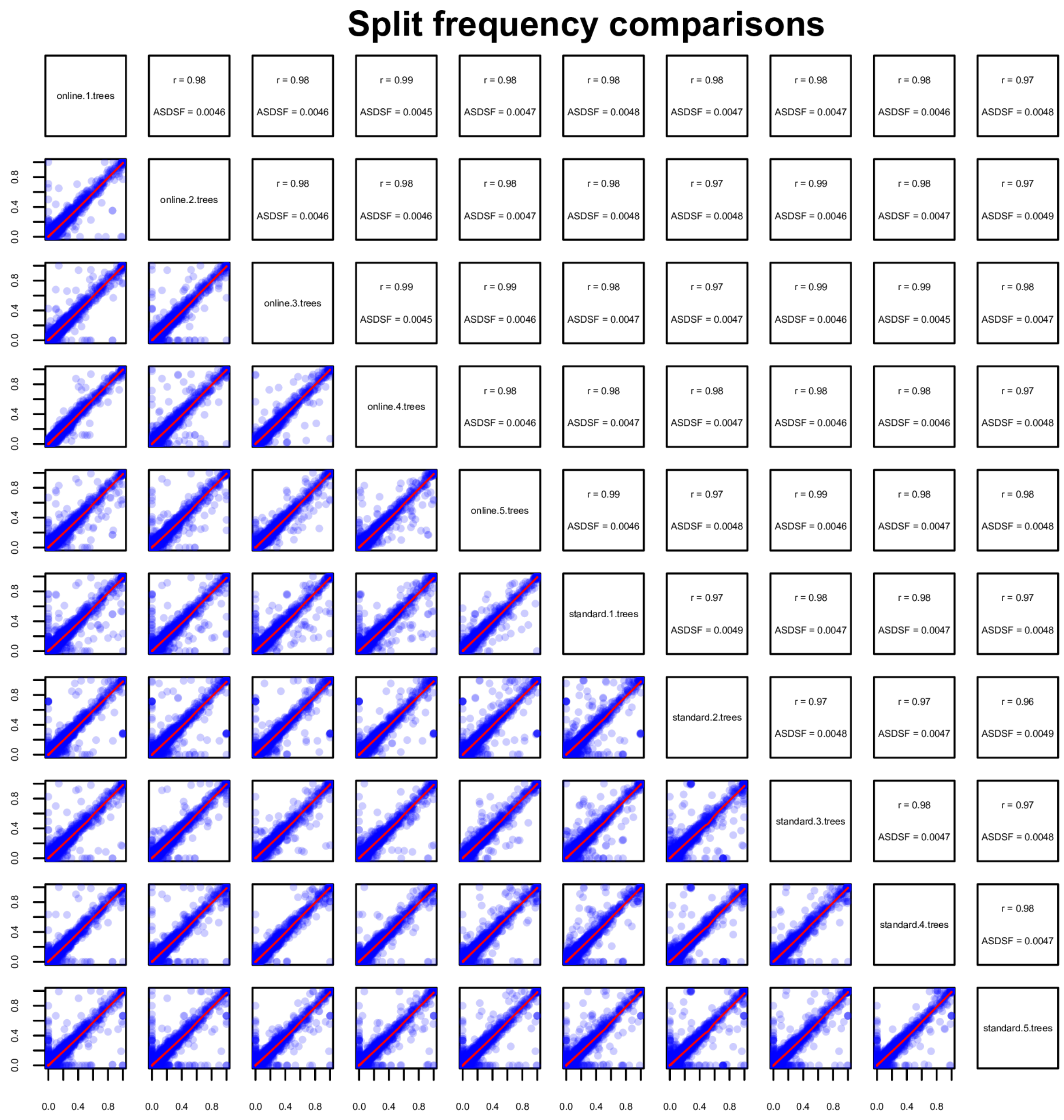

Figure S9: Comparison of split (clade) frequencies in tree samples from five standard and five online analyses of data from epi week 2 of 2015 of West African Ebola virus epidemic. For each pair of chains, the frequencies of clades in the different chains are plotted against each other in the plots below the diagonal of the figure, and the correlation and the average standard deviation of split frequencies (ASDSF) of the pair are shown above the diagonal. The diagonal entries correspond to the ten independent replicates and indicate the specific pairwise comparisons that are made below and above the diagonal.

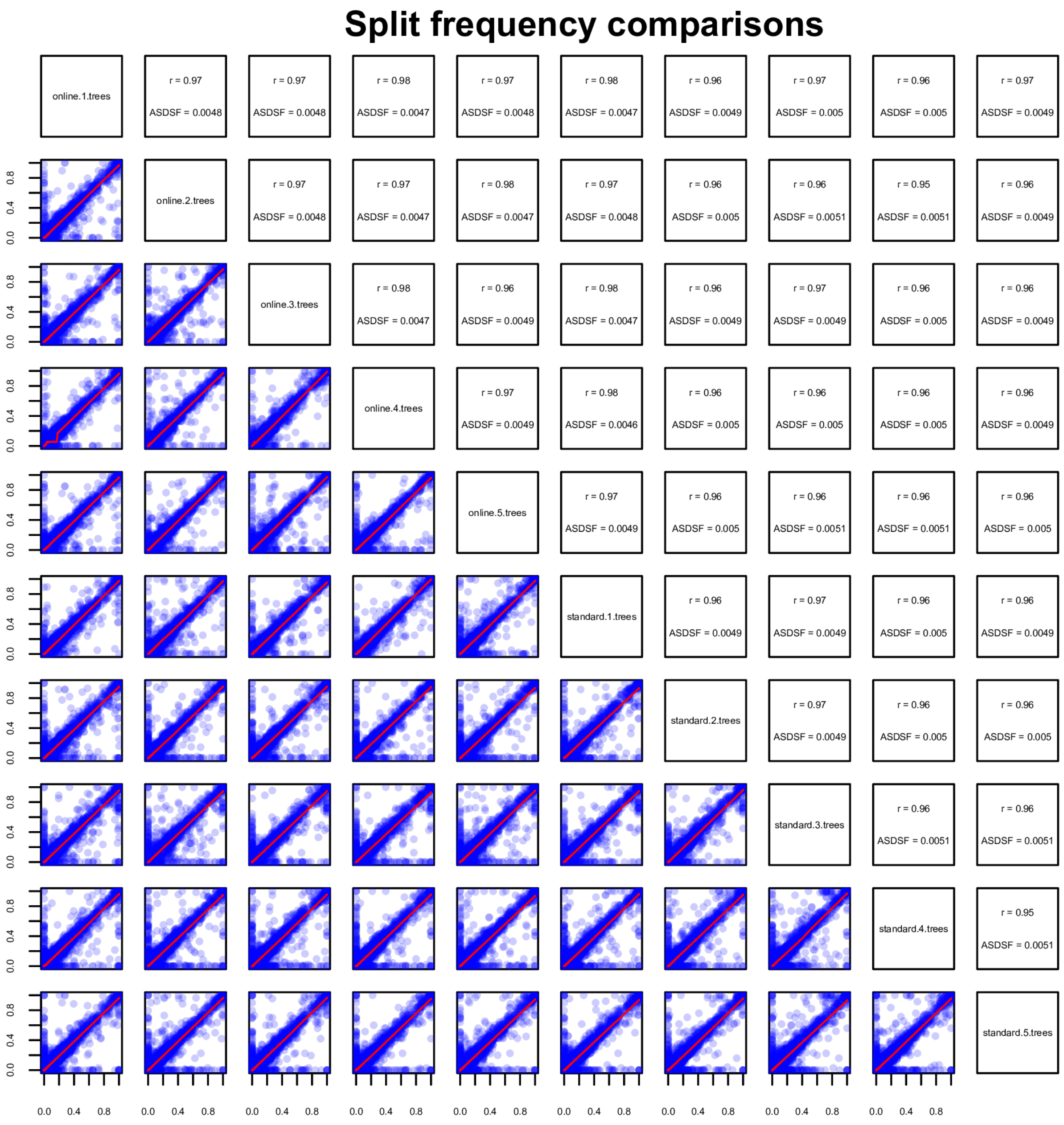

Figure S10: Comparison of split (clade) frequencies in tree samples from five standard and five online analyses of data from epi week 42 of 2015 of West African Ebola virus epidemic. For each pair of chains, the frequencies of clades in the different chains are plotted against each other in the plots below the diagonal of the figure, and the correlation and the average standard deviation of split frequencies (ASDSF) of the pair are shown above the diagonal. The diagonal entries correspond to the ten independent replicates and indicate the specific pairwise comparisons that are made below and above the diagonal.

2.3 Genetic Divergence of New Sequences from Existing Sequences

For each epi week of the Ebola virus example for which we computed updated inferences through an online analysis, the average burn-in was less than or equal to one million iterations. Here, we examine for each time point the degree of divergence between the new sequences that are being incorporated into the online analysis and the sequences that already exist in the tree. In particular, we compute the genetic distance between each new sequence and the closest sequence to it that is already present in the tree. We refer to this distance as the "divergence" and show the mean divergence (with standard deviation in parentheses) and maximal divergence for each time point in Table S1. While the varying degrees of divergence we find here do not correspond to notable differences in burn-in in this example, it is important in general to be mindful of the potential impact of divergence in online analyses.

	Sequences		Divergence		Online analysis	
Data	Total	Added	Mean	Max	Burn-in (G)	Burn-in (ESS)
2014, Epi week 26	158	13	0.00011 (0.00015)	0.00053	<0.1 (<0.1)	<0.1 (<0.1)
2014, Epi week 31	240	8	0.00016 (0.00009)	0.00026	<0.1 (<0.1)	0.4 (0.9)
2014, Epi week 42	706	32	0.00012 (0.00010)	0.00042	0.6 (0.9)	1.0 (1.0)
2015, Epi week 2	1072	24	0.00021 (0.00058)	0.00014	0.6 (0.5)	0.4 (0.5)
2015, Epi week 42	1610	2	0.00005 (0)	0.00005	<0.1 (<0.1)	0.6 (1.3)

Table S1: Divergence of new sequences from existing sequences.

References

Drummond AJ, Ho SYW, Phillips MJ, Rambaut A. 2006. Relaxed phylogenetics and dating with confidence. *PLoS Biol.* 4:e88.

Lakner C, van der Mark P, Huelsenbeck JP, Larget B, Ronquist F. 2008. Efficiency of Markov chain Monte Carlo tree proposals in Bayesian phylogenetics. *Syst. Biol.* 57:86–103.

Ronquist F, Huelsenbeck JP, Teslenko M. 2011. Draft MrBayes version 3.2 manual: tutorials and model summaries.

Warren DL, Geneva AJ, Lanfear R. 2017. RWTY: (R We There Yet): an R package for examining convergence of Bayesian phylogenetic analyses. *Mol. Biol. Evol.* 34:1016–1020.
